# Antibiotic prophylaxis for percutaneous nephrolithotomy: An updated systematic review and meta-analysis

**DOI:** 10.1371/journal.pone.0267233

**Published:** 2022-04-15

**Authors:** Hae Do Jung, Kang Su Cho, Young Joon Moon, Doo Yong Chung, Dong Hyuk Kang, Joo Yong Lee

**Affiliations:** 1 Department of Urology, Wonkwang University Sanbon Hospital, Wonkwang University School of Medicine, Gunpo, Republic of Korea; 2 Department of Urology, Gangnam Severance Hospital, Urological Science Institute, Yonsei University College of Medicine, Seoul, Korea; 3 Department of Urology, Severance Hospital, Urological Science Institute, Yonsei University College of Medicine, Seoul, Korea; 4 Department of Urology, Inha University Hospital, Inha University School of Medicine, Incheon, Korea; 5 Center of Evidence Based Medicine, Institute of Convergence Science, Yonsei University, Seoul, Korea; Nitte University, INDIA

## Abstract

**Introduction:**

A single dose of preventive antibiotics is known to be sufficient to reduce the rate of infection-related complications in percutaneous nephrolithotomy (PCNL). However, some studies reported that the extended dose showed lower complications for high-risk groups. Therefore, we performed a systematic review and meta-analysis comparing single- and extended-dose antibiotic prophylaxis for PCNL.

**Materials and methods:**

Relevant studies that compared single- and extended-dose antibiotic prophylactic therapies were identified. Articles were selected from PubMed, EMBASE, KoreaMed, and Google Scholar up to September 2021. Fever and systemic inflammatory response syndrome (SIRS) were compared by meta-analysis. A subgroup analysis was performed according to the degree of risk to the patient.

**Results:**

A total of 10 articles were included in this study. There were no significant differences between single dose and extended dose in the rate of fever [p = 0.93, OR = 0.96, 95% confidence interval (CI) 0.44–2.13, I^2^ = 64%]. Extended dose showed lower rate of SIRS compared to single dose (p = 0.0005, OR = 1.81, 95% CI 1.30–2.53, I^2^ = 53%); in the subgroup analysis, extended dose also showed lower rates of SIRS compared to single dose in high-risk patients (p <0.0001, OR = 3.53, 95% CI 1.91–6.54, I^2^ = 36%).

**Conclusions:**

The results of our meta-analysis showed that single-dose antibiotic prophylaxis can be effective for PCNL, but extended-dose antibiotics can be required in high-risk patients to reduce post-PCNL infection-related complications.

## Introduction

Percutaneous nephrolithotomy (PCNL) has been the standard treatment for large renal stones since the first case reports by Fernstorm and Johnson in 1976 [1]. Since then, advances have been made in renal access, optics, radiology, and lithotripsy. The incidence rates of infection-related complications, such as fever and sepsis, after PCNL are 10.8% and 0.5%, respectively [2]. To reduce the incidence of such complications, the European Association of Urology (EAU) Guidelines on Urolithiasis has suggested the use of a single-dose prophylactic antibiotics [2]. The American Urological Association (AUA) guideline recommends antibiotic prophylaxis for PCNL, with the administration of perioperative antibiotic therapy within 60 minutes of the procedure [3].

However, some studies have reported that an extended dose of prophylactic antibiotics showed lower rates of infection for high-risk groups vulnerable to post-PCNL complications compared to a single dose [4, 5]. Moreover, whether the administration of preoperative versus perioperative antibiotic prophylaxis is better remains controversial.

In its 2014 global monitoring report on antibiotic resistance, the World Health Organization (WHO) noted that the increased resistance of major bacteria to cephalosporins and fluoroquinolones was a serious health problem worldwide [6]. Considering such antibiotic resistance, appropriate short-term antibiotic therapy is recommended. Therefore, we conducted a meta-analysis comparing the effects of single- and extended-dose antibiotic prophylaxis for PCNL.

## Materials and methods

### Inclusion criteria

The inclusion criteria of this study were as follows: (a) patients with renal stones who underwent PCNL, (b) comparison of single- and extended-dose antibiotic prophylaxis for PCNL, and (c) outcome measures including fever and systematic inflammatory response syndrome (SIRS). A published study was excluded if it was not available in full text. This report was prepared in compliance with the Preferred Reporting Items for Systematic Reviews and Meta-Analyses statement (S1 Table) [7]. This systematic review was exempt from consideration by the ethics committee or institutional review board, as systematic reviews and meta-analyses do not require prior approval.

### Search strategy

A systematic review was conducted to identify relevant, comparative articles that described the prophylactic use of single and extended doses of antibiotics for PCNL in PubMed, EMBASE, KoreaMed, and Google Scholar up to September 2021.

Search strategies were established to include medical subject headings keywords, such as “kidney calculi,” “urolithiasis,” “percutaneous nephrolithotomy,” “PCNL,” “antibiotic prophylaxis,” and combinations of these search terms (S2 Table).

### Study selection and data extraction

Two researchers screened the titles and abstracts of articles that were independently identified by the search strategy to exclude irrelevant studies. They also assessed the full text of the articles for relevance. The most relevant articles were extracted from each study, and information such as author, year of publication, country, study design, patient characteristics (e.g., high risk), and treatments were recorded, as well as outcome variables such as “fever” and “SIRS”.

### Study quality assessment

We used the Cochrane Risk of Bias (ROB) tool for randomized control trial (RCT) and the methodological index for non-randomized studies (MINORS) for non-randomized trials.

Quality of evidence was graded independently by our researchers (HDJ & DHK) using the Scottish Intercollegiate Guidelines Network (SIGN) methodology checklist. The SIGN checklist was used to assess the quality of various types of research, including systematic reviews and meta-analyses, randomized controlled trials (RCTs), cohort studies, case-control studies, diagnostic studies, and economic studies. All disagreements regarding the quality assessment results were resolved after discussion with a third reviewer (JYL).

### Statistical analysis

The odds ratios (ORs) and 95% confidence intervals (CIs) were calculated and reported. The chi-square test with p-values less than 0.05 was used to evaluate statistical heterogeneity, and the I^2^ statistic was used to quantify heterogeneity [8]. If the reported I^2^ was less than 50%, we applied the fixed-effects model; otherwise, the random-effects model was used. The Higgins I^2^ statistic was calculated as follows:

$$I^{2}=\frac{Q-df}{Q}\times 100\%$$

where "Q" is the Cochrane heterogeneity statistic and "df" is the degree of freedom. All meta-analyses were performed using Review Manager, version 5.4.1 (RevMan, Copenhagen: The Nordic Cochrane Center, The Cochrane Collaboration, 2020).

A subgroup analysis was performed in three patient groups according to the degree of risk to the patient. If the patient had hydronephrosis or a stone size greater than or equal to 2 cm, the patient was classified as "high risk"; a patient was categorized as "low risk" in the absence of hydronephrosis or with a stone size less than 2 cm. Patients were considered "not specified" if the risk could not be determined. This systematic review is registered in PROSPERO, CRD 42022297928.

## Results

### Eligible studies

**Fig 1:**
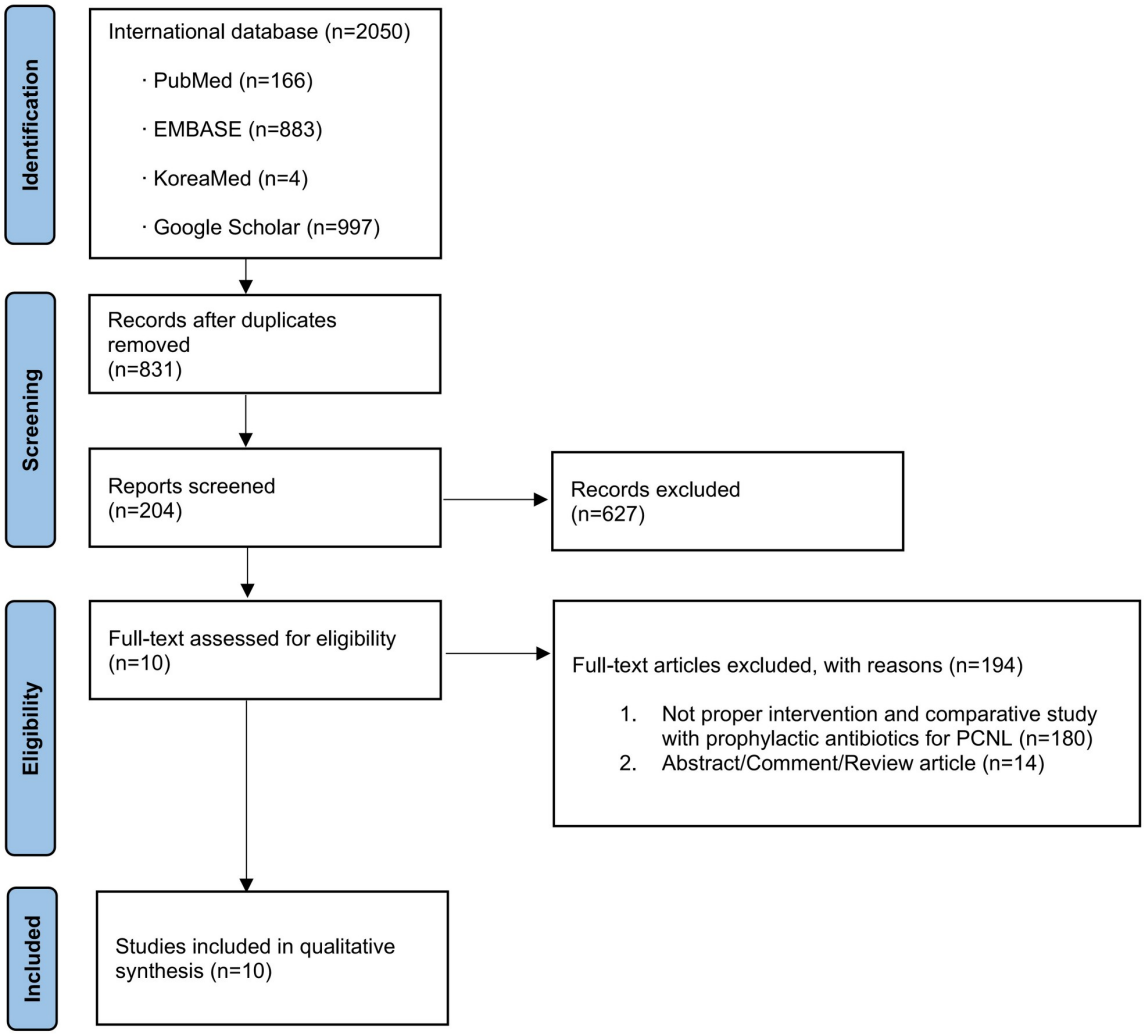
Study flow chart.

A total of 831 studies were identified for potential inclusion in the meta-analysis. After a full-text review, 10 articles were identified as relevant for this study and selected for inclusion in the meta-analysis (Fig 1) [4, 5, 9–16].

### Characteristics of the included studies

**Fig 2:**
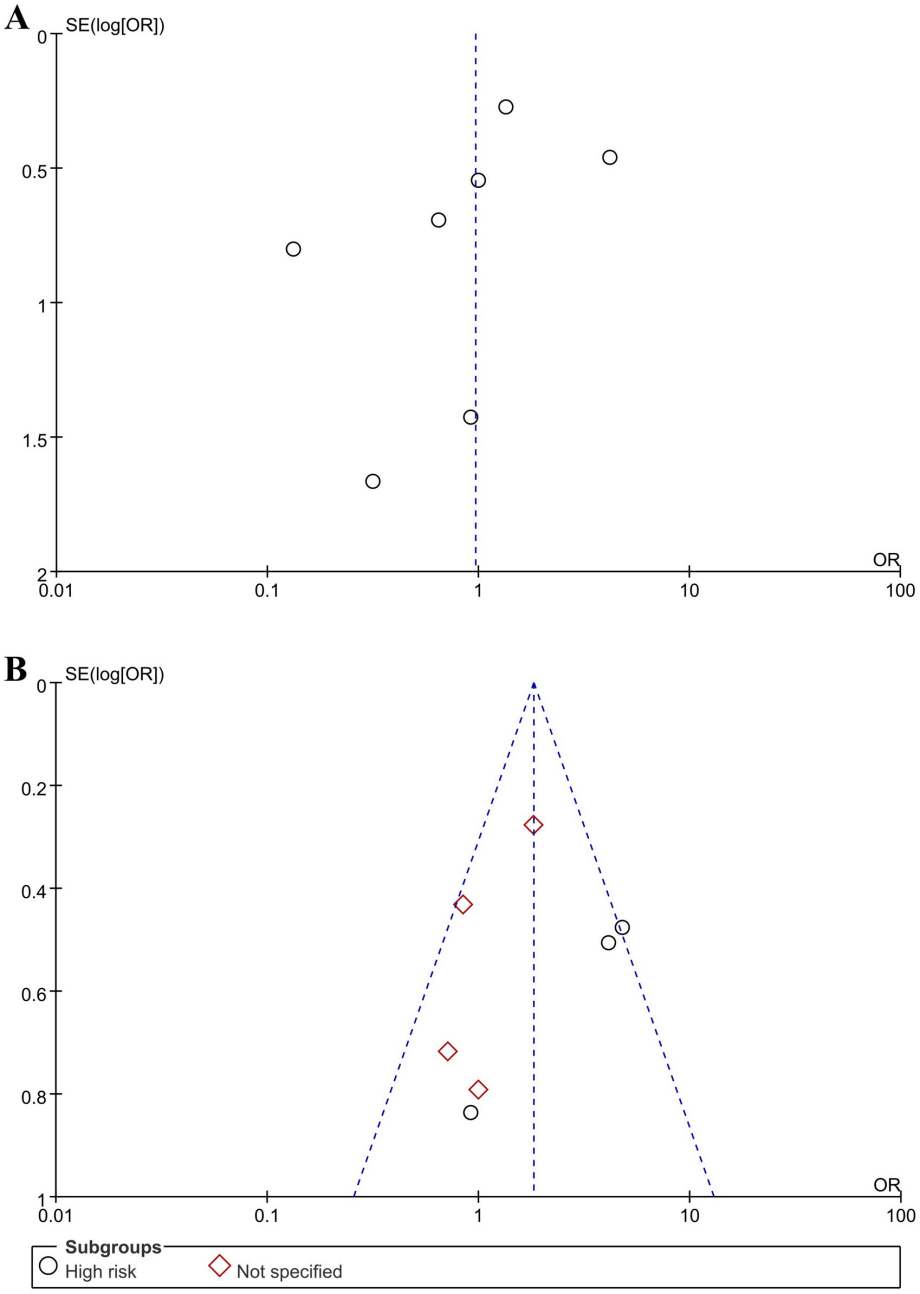
Funnel plot. (A) Single dose vs. extended dose in patients with fever; (B) Single dose vs. extended dose in patients with SIRS.

**Fig 3:**
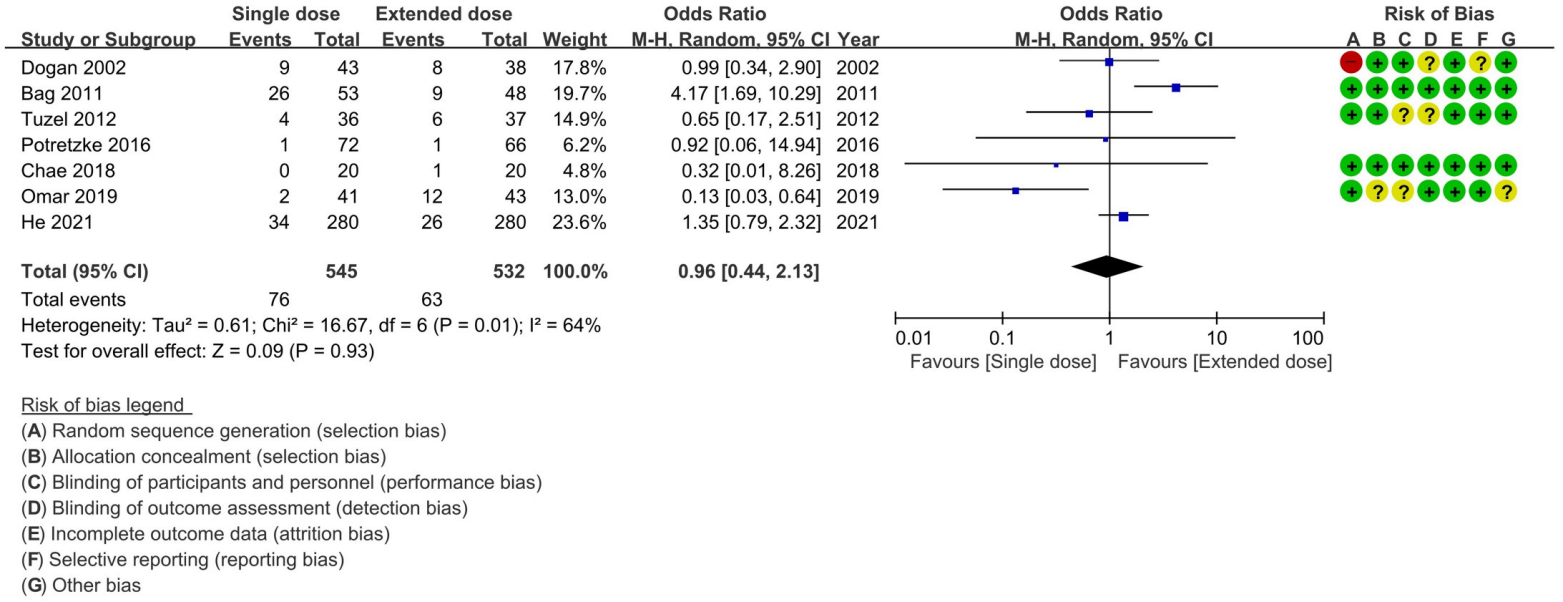
Forest plot. Single dose vs. extended dose in patients with fever.

**Fig 4:**
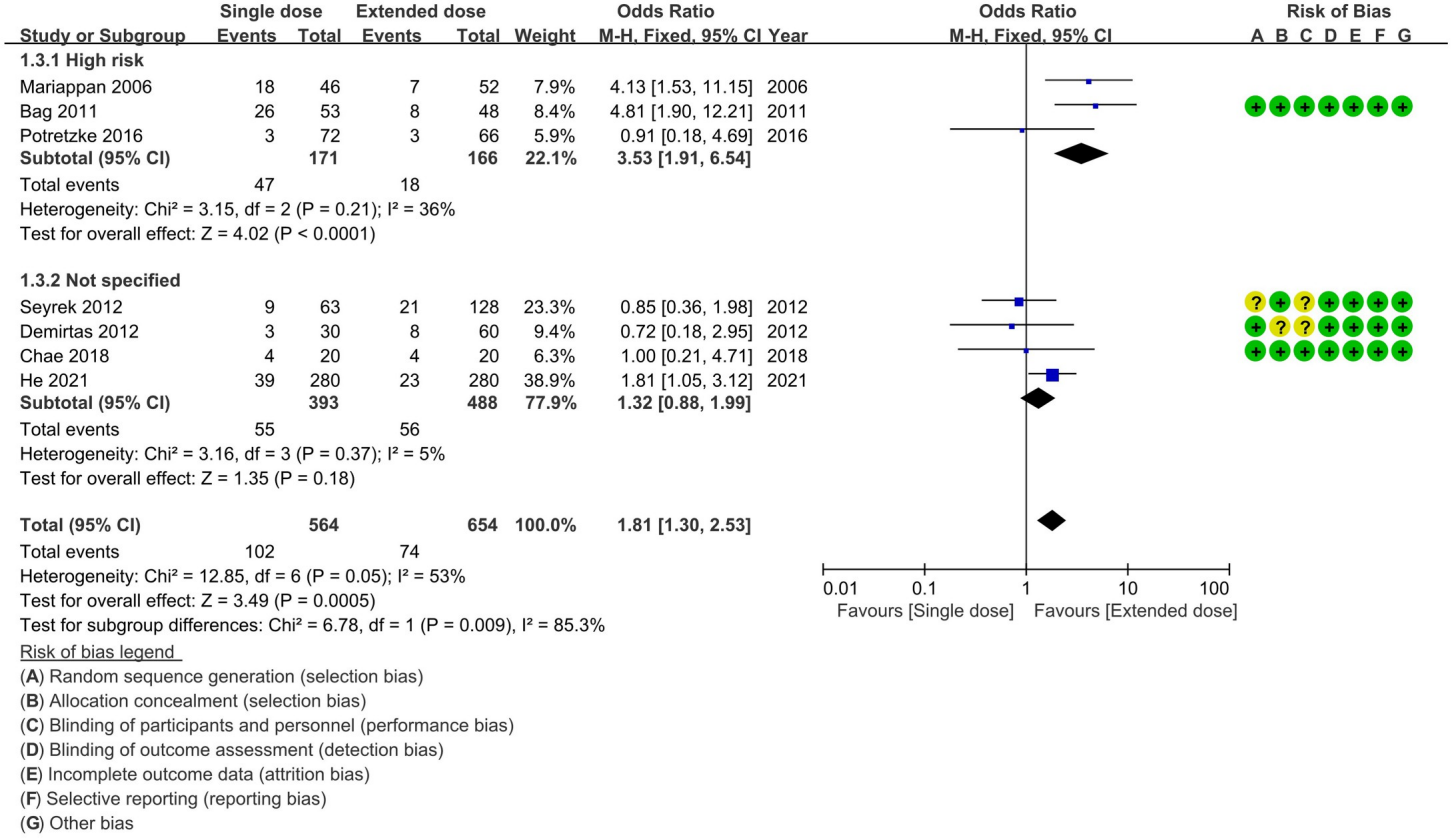
Forest plot. Single dose vs. extended dose in patients with SIRS.

The characteristics of the 10 included studies are shown in Table 1 [4, 5, 9–16]. These comparative studies described patients who underwent antibiotic prophylaxis for PCNL for renal stones. The included studies were published between November 2002 and March 2021. Five studies were performed in Europe (four studies from Turkey and one study from the UK) [5, 9, 10, 15, 16]. Three studies were performed in Asia (China, India, and South Korea) [4, 11, 12], and one study was performed in the U.S [14]. Additionally, one study was conducted in Egypt [13]. Four studies were selected based on the inclusion of a high-risk patient group [4, 5, 14, 16]; six other studies were chosen but did not specify the patient group [9–13, 15]. Four studies were selected based on the use of an “extended preop” [4, 5, 12, 14], whereas six other studies were chosen due to the use of an “extended periop” [9–11, 13, 15, 16]. “Extended periop” refers to extended preoperative and postoperative antibiotic use. The results of quality assessment of the included studies are shown in Table 1, which were found to be acceptable. Seven studies were indicated as 1+, two studies were indicated as 2+, and one study was indicated as 2-. Funnel plots of the meta-analyses are shown in Fig 2. There was little publication bias in most of the included studies. The ROB for RCTs is shown in Figs 3 and 4. The MINORS scores for non-RCTs are displayed in Table 2. All studies were considered reasonable.

**Table 1:** Characteristics of included studies.

Citation	Country	Study design	Procedure	Inclusion criteria	Patients, n	Mean age, years ± SD	Quality assessment
He et al. 2021	China	Retrospective	Single-dose Preop	Not specified	280	53.2±15.4	2+
			Extended-dose Preop		280	54.7±14.1	
Omar et al. 2019	Egypt	RCT	Single-dose Preop	Not specified	41	51±12	1+
			Extended-dose Periop		43	50±11	
Chae et al. 2018	Korea	RCT	Single-dose Preop	Not specified	20	56.7±10.1	1+
			Extended-dose Periop		20	54.0±11.1	
Potretzke et al. 2016	USA	Retrospective	Single-dose Preop	High risk	72	61	2-
			Extended-dose Preop		66	59.5	
Tuzel et al. 2012	Turkey	RCT	Single-dose Preop	High risk	36	43.5	1+
			Extended-dose Periop		37	44.7	
Demirtas et al. 2012	Turkey	RCT	Single-dose Preop	Not specified	30	43.9±14.03	1+
			Extended-dose Periop		60		
Seyrek et al. 2012	Turkey	RCT	Single-dose Preop	Not specified	63	43.8±14.3	1+
			Extended-dose Periop		128		
Bag et al. 2011	India	RCT	Single-dose Preop	High risk	53	40.4±13.0	1+
			Extended-dose Preop		48	39.2±12.1	
Mariappan et al. 2006	UK	Prospective	Single-dose Preop	High risk	46	53.1	2+
			Extended-dose Preop		52	55.5	
Dogan et al. 2002	Turkey	RCT	Single-dose Preop	Not specified	43	41.4	1+
			Extended-dose Periop		38	37.5	

RCT, randomized controlled trials; SD, standard deviation. Quality assessment was indicated by the Scottish Intercollegiate Guidelines Network (SIGN) checklist. 1+ means well-conducted RCT with a low risk of bias. 1- means RCT with a high risk of bias. 2+ means well-conducted cohort studies with a low risk of bias. 2- means cohort studies with a high risk of bias.

**Table 2:** MINORS score in non-randomized studies included in the review.

	He et al. 2021	Potretzke et al. 2016	Mariappan et al. 2006
**A clearly stated aim**	2	2	2
**Inclusion of consecutive samples**	2	2	2
**Prospective collection of data**	0	0	2
**Endpoints appropriate to the aim of the study**	2	2	2
**Unbiased assessment of the study endpoint**	0	0	0
**Follow-up period appropriate to the aim of the study**	2	2	2
**Loss to follow-up less than 5%**	2	2	2
**Prospective calculation of the study size**	0	0	0
**An adequate control group**	2	2	2
**Contemporary groups**	2	2	2
**Baseline equivalence of groups**	2	2	2
**Adequate statistical analyses**	2	2	2
**Total**	18	18	20

MINORS, methodological index for non-randomized studies. The items are scored as 0 (not reported), 1 (reported but inadequate), or 2 (reported and adequate). The global ideal score is 16 for non-comparative studies and 24 for comparative studies.

### Heterogeneity assessment

The results of heterogeneity test showed that conspicuous heterogeneities were discerned in the analysis of complication rates. For fever, there were some heterogeneities (p = 0.01, I^2^ = 64%). Random-effects models were used to compare the complication rates of fever between the use of single- and extended-dose antibiotics (Fig 3). For SIRS, there was some heterogeneity (p = 0.05, I^2^ = 53%), but the subgroup ("high risk" and "not specified") analysis revealed that there were few heterogeneities (p = 0.21, I^2^ = 36% and p = 0.37, I^2^ = 5%, respectively). Therefore, fixed-effects models were used to compare the complication rates between the use of single- and extended-dose antibiotics (Fig 4).

### Complication rates

Fever was compared between the use of single and extended doses of antibiotics in seven studies [4, 10–14, 16], but no significant differences were observed (p = 0.93, OR = 0.96, 95% CI 0.44–2.13, I^2^ = 64%) (Fig 3). Risk classification subgroup analysis was not performed, as there were no statistically significant differences between the broader groups.

SIRS was compared between the use of single and extended doses of antibiotics in seven studies [4, 5, 9, 11, 12, 14, 15]. A risk classification subgroup analysis was performed, revealing a significant subgroup effect (p = 0.009). Three studies involved high-risk patient groups [4, 5, 14], whereas four studies did not specify the patient group [9, 11, 12, 15]. The use of an extended antibiotic dose showed lower rates of SIRS than the use of a single dose (p = 0.0005, OR = 1.81, 95% CI 1.30–2.53, I^2^ = 53%). The results of subgroup analysis revealed lower rates of SIRS in high-risk patients who were administered extended dose of antibiotics compared to those administered single dose (p <0.0001, OR = 3.53, 95% CI 1.91–6.54, I^2^ = 36%), but there were no significant differences between patients in the studies that did not specify the patient group (p = 0.18, OR = 1.32, 95% CI 0.88–1.99, I^2^ = 5%) (Fig 4).

## Discussion

Recently, Yu *et al*. published a systematic review and meta-analysis on the use of antibiotic prophylaxis for PCNL [17]. They analyzed 13 studies and reported the effects of extended dose of preoperative prophylactic antibiotics, single- and extended-dose postoperative prophylactic antibiotics, and single dose of antibiotics administered before anesthesia. The use of an extended dose of preoperative antibiotics resulted in a lower sepsis rate compared to the use of a single dose (p <0.00001, OR = 0.31, 95% CI 0.20–0.50, I^2^ = 30%). On the other hand, the use of an extended dose of postoperative antibiotics was not superior to a single dose administered prior to anesthesia in terms of the sepsis rate (p = 0.49, RR = 1.19, 95% CI 0.72–1.97, I^2^ = 0%). These authors concluded that extended-dose preoperative prophylactic antibiotics lowered the risk of postoperative sepsis and fever. However, in their study, the reason for the lower sepsis rate in patients administered extended dose of preoperative antibiotics may result from the fact that four of the five studies used in their meta-analysis were conducted in high-risk patients.

There are no recommendations for the use of antibiotic prophylaxis for PCNL in high-risk patients in either the EAU Guidelines on Urolithiasis and Urological Infections or the AUA guidelines [2, 3]. Recently, Sur *et al*. conducted an RCT on the use of 2 days or 7 days of preoperative antibiotics for PCNL in patients at moderate to high risk of infection [18]. These authors found that 7 days was superior to 2 days for reducing sepsis (p = 0.031, OR = 0.31, 95% CI 1.11–8.93). Chew *et al*. also reported that the use of extended-dose preoperative antibiotics offered no advantage in low-risk patients (negative preoperative urine cultures and no urinary drain) [19]. In their study, the sepsis rate was not statistically different between the group receiving an extended dose of preoperative antibiotics and the control group (p = 1.0, mean difference –0.020, 95% CI –0.163–0.122). Additionally, other parameters of infection (intensive care hospitalization, fever, hypotension, and leukocytosis) were similar between the groups.

We analyzed the effectiveness of preventive antibiotics for PCNL by distinguishing high-risk patients in a subgroup analysis. The use of an extended dose of antibiotics resulted in lower rates of SIRS compared to a single dose in high-risk patients (p <0.0001, OR = 3.53, 95% CI 1.91–6.54, I^2^ = 36%), but not in non-specified patients (p = 0.18, OR = 1.32, 95% CI 0.88–1.99, I^2^ = 5%).

To prevent post-PCNL infections, a urine culture should be obtained prior to PCNL, and appropriate antibiotics should be prescribed according to the results of the urine culture, which should be sterile preoperatively [3]. However, urinary tract infections and sepsis can occur after PCNL, even if a sterile urine is confirmed prior to the procedure [20]. For these reasons, antibiotic prophylaxis is necessary for PCNL. Furthermore, antibiotic prophylaxis should be used for antimicrobial stewardship, which is defined as "an organizational or healthcare system-wide approach to promoting and monitoring judicious use of antimicrobial to preserve their future effectiveness" [21]. In other words, antimicrobial stewardship means using appropriate dose of antibiotics when necessary, and it aims to optimize clinical outcomes and cost-effectiveness while minimizing the emergence of resistant bacterial strains through adherence to local, national, and international guidelines [21–23]. Antimicrobial resistance is the ability of microorganisms to remain viable even with the use of antimicrobial agents, and is considered a natural phenomenon that occurs via genetic mutation within the bacteria [22]. However, the ongoing rapid evolution of resistance in bacterial uropathogens is occurring worldwide [6, 24]. In a global report on antibiotic resistance by the WHO in 2014 [25], the resistance of *Escherichia coli* to third-generation cephalosporins was reported to be 87% in Africa, 68% in the Americas, 94% in the Eastern Mediterranean, 82% in Europe, 95% in Southeast Asia, and 77% in the Western Pacific. Resistance of *E*. *coli* to fluoroquinolones was reported to be 98% in Africa, 60% in the Americas, 91% in the Eastern Mediterranean, 48% in Europe, 89% in Southeast Asia, and 94% in the Western Pacific. In addition, despite repeated warnings from the WHO, there is a severe shortage of new antibiotics to cope with the emerging antibiotic resistance crisis [22]. The U.S. Centers for Disease Control and Prevention has estimated that more than 2 million people are affected by antibiotic-resistant infections every year in the United States, with at least 23,000 deaths [26]. In Europe, the overall economic burden due to antibiotic resistance was estimated at €1.5 billion, with more than €900 million related to hospital costs [27].

To reduce antibiotic resistance, using as few antibiotics as possible is essential. The overuse of antibiotics reveals an obvious connection with the development of resistance [22, 28]. Through the excessive use of a particular antibiotic, bacteria can gain resistance to it [16]. As the prevalence of resistance to particular antibiotics increases in the global population, the possible failure of an empirical antibiotic outweighs its benefits. Such a failure can cause postoperative infections, resulting in the use of more antibiotics [16, 29]. Therefore, using a single dose of antibiotics can prevent the waste of medical resources and decrease the cost and duration of hospital stays for PCNL. However, in high-risk patients who are susceptible to post-PCNL infections, it may be better to consider the use of extended-dose antibiotics.

The current study had some limitations. First, it only included 10 studies. Seven of them were RCTs, one was a prospective study, and two were retrospective studies; but they were included as a whole. If additional studies are published in the future, the results reported here can updated. Second, it was not possible to classify the types of antibiotics used (e.g., narrow-spectrum vs. broad-spectrum) that could impact the outcomes. Since antibiotic resistance varies by country and region, it is not possible to analyze the effects of the same antibiotics for PCNL. The use of antibiotics is recommended according to local antimicrobial susceptibility information, clinical settings, and a patient’s risk factors for antibiotic resistance [30]. Therefore, the results of this study can be applied to real clinical settings and high-risk patients, as the study recommends careful attention be paid to the use of a single dose of antibiotics. Third, the resistance of cultured microorganisms may impact the outcomes (e.g., multidrug-resistant organisms). However, we could not analyze the impact of isolated microorganisms due to variable microorganisms in the included studies. Fourth, we could not analyze the impact of differences in the duration of extended perioperative prophylactic antibiotics, as the duration of antibiotics in the included studies varied too much. The duration of empiric antibiotic treatment may also be impacted by the type of antibiotic used and the organisms involved. These factors could not be analyzed due to variable antibiotics and the absence of culture reports. Fifth, we did not distinguish extended preoperative from extended perioperative (both preoperative and postoperative) prophylactic antibiotics. The reason is that there were three studies of high-risk patients that used extended preoperative prophylactic antibiotics, while there was only one study of high-risk patients that used extended perioperative prophylactic antibiotics.

## Conclusions

A single dose of antibiotics administered prophylactically for PCNL can be effective and sufficient; however, in patients at high risk for post-PCNL infections, the use of extended dose of antibiotics may be required. Therefore, future prospective randomized trials with large sample sizes should be performed on this matter.

## Supporting information

S1 Table PRISMA checklist. (DOCX)

S2 Table Search strategy. (DOCX)
