# Supplementary material for: Antibiotic prophylaxis for percutaneous nephrolithotomy: An updated systematic review and meta-analysis
Source: PLoS One. 2022 Apr 15;17(4):e0267233. doi: 10.1371/journal.pone.0267233 (PMC9012355; doi:10.1371/journal.pone.0267233)
Supplement: S2 Table — (DOCX) [file pone.0267233.s002.docx]

**S2 Table. Search strategy**

| **PubMed** | | | |
| --- | --- | --- | --- |
| Search | Query | Items found | |
| 1 | kidney calculi | 24,658 | |
| 2 | urolithiasis | 43,273 | |
| 3 | #1 OR #2 | 44,369 | |
| 4 | percutaneous nephrolithotomy | 4,556 | |
| 5 | PCNL | 2,437 | |
| 6 | #4 OR #5 | 4,839 | |
| 7 | #3 AND #6 | 3,481 | |
| 8 | antibiotic prophylaxis | 24,377 | |
| 9 | #7 AND #8 | 166 | |
| **EMBASE** | | | |
| Search | Query | Items found | |
| 1 | kidney calculi | 40,886 | |
| 2 | urolithiasis | 76,222 | |
| 3 | #1 OR #2 | 81,250 | |
| 4 | percutaneous nephrolithotomy | 10,556 | |
| 5 | PCNL | 6,046 | |
| 6 | #4 OR #5 | 11,221 | |
| 7 | #3 AND #6 | 8,304 | |
| 8 | antibiotic prophylaxis | 40,317 | |
| 9 | #7 AND #8 | 883 | |
| **KoreaMed** | | | |
| Search | Query | Items found | |
| 1 | kidney calculi | 152 | |
| 2 | urolithiasis | 274 | |
| 3 | #1 OR #2 | 274 | |
| 4 | percutaneous nephrolithotomy | 103 | |
| 5 | PCNL | 39 | |
| 6 | #4 OR #5 | 105 | |
| 7 | #3 AND #6 | 5 | |
| 8 | antibiotic prophylaxis | 157 | |
| 9 | #7 AND #8 | 4 | |
| **Google Scholar** | | | |
| Search | Query | Items found | |
| 1 | ("kidney calculi" OR "urolithiasis") AND ("percutaneous nephrolithotomy" OR "PCNL") AND "antibiotic prophylaxis" | | 997 |
